# A binuclear cobalt(II) complex of an NO_3_-donor Schiff base derived from 3-carboxyl­salicylaldehyde and 2-nitro­aniline

**DOI:** 10.1107/S1600536808042487

**Published:** 2008-12-20

**Authors:** Zhao-Wen Yu, Ling Chang, Peng Sun, Min-Hui He

**Affiliations:** a Institute of Molecular and Crystal Engineering, College of Chemistry and Chemical Engineering, Henan University, Kaifeng 475001, Henan, People’s Republic of China

## Abstract

In the crystal structure of the centrosymmetric title complex, bis­{μ-3-[(2-nitro­phen­yl)imino­meth­yl]-2-oxidobenzoato}dicobalt(II), [Co_2_(C_14_H_8_N_2_O_5_)_2_], in which the ligand is 3-[(2-nitro­phen­yl)imino­meth­yl]-2-oxidobenzoate, a Schiff base synthesized from 2-nitro­aniline with 3-carboxyl­salicyl­aldehyde, the two cobalt(II) ions in the mol­ecular unit are bridged by two phenolate O atoms of the ligands. Each metal centre has a distorted square-planar geometry. In the crystal structure, mol­ecules are linked by Co⋯O inter­actions involving the nitro O atoms, forming a two-dimensional network. There are also C—H⋯O and π–π stacking inter­actions [centroid–centroid distances of 3.5004 (2), 3.6671 (2) and 3.6677 (2) Å] between adjacent benzene rings of the two-dimensional networks, leading to the formation of a three-dimensional framework.

## Related literature

For binuclear cobalt(II) complexes of Schiff base ligands, see: Adams *et al.* (2002 ▶); Tone *et al.* (2007 ▶). For the design of mol­ecular solids, see: Zheng *et al.* (2003 ▶). For bond-valence parameters, see: Brown & Altermatt (1985 ▶). For luminescence emission, see: Li *et al.* (2008 ▶).
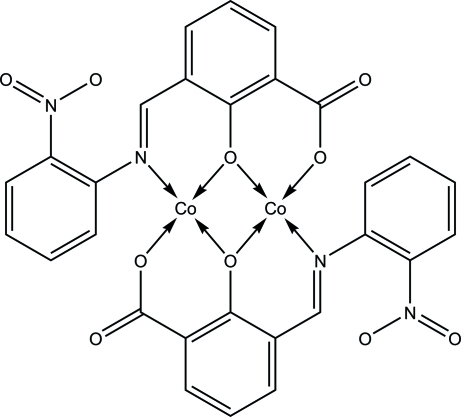


## Experimental

### 

#### Crystal data

[Co_2_(C_14_H_8_N_2_O_5_)_2_]
*M* *_r_* = 686.31
Monoclinic, 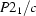

*a* = 8.3398 (6) Å
*b* = 11.0454 (8) Å
*c* = 13.3681 (9) Å
β = 99.6040 (10)°
*V* = 1214.16 (15) Å^3^
*Z* = 2
Mo *K*α radiation
μ = 1.44 mm^−1^
*T* = 296 (2) K
0.21 × 0.16 × 0.11 mm

#### Data collection

Bruker APEXII CCD area-detector diffractometer
Absorption correction: multi-scan (*SADABS*; Bruker, 2005 ▶) *T*_min_ = 0.752, *T*_max_ = 0.858
6134 measured reflections
2132 independent reflections
1536 reflections with *I* > 2σ(*I*)
*R*_int_ = 0.045

#### Refinement

*R*[*F* ^2^ > 2σ(*F* ^2^)] = 0.045
*wR*(*F* ^2^) = 0.123
*S* = 1.00
2132 reflections
199 parameters
H-atom parameters constrained
Δρ_max_ = 0.63 e Å^−3^
Δρ_min_ = −0.34 e Å^−3^

### 

Data collection: *APEX2* (Bruker, 2005 ▶); cell refinement: *SAINT* (Bruker, 2005 ▶); data reduction: *SAINT*; program(s) used to solve structure: *SHELXS97* (Sheldrick, 2008 ▶); program(s) used to refine structure: *SHELXL97* (Sheldrick, 2008 ▶); molecular graphics: *SHELXTL* (Sheldrick, 2008 ▶); software used to prepare material for publication: *SHELXTL* and *PLATON* (Spek, 2003 ▶).

## Supplementary Material

Crystal structure: contains datablocks I, global. DOI: 10.1107/S1600536808042487/su2076sup1.cif

Structure factors: contains datablocks I. DOI: 10.1107/S1600536808042487/su2076Isup2.hkl

Additional supplementary materials:  crystallographic information; 3D view; checkCIF report

## Figures and Tables

**Table:** 

Co1—O3	1.936 (3)
Co1—N1	1.947 (4)
Co1—O2^i^	1.874 (3)
Co1—O3^i^	1.929 (3)

**Table:** 

Co1⋯O4	2.807 (4)
Co1⋯O5^ii^	2.867 (5)

**Table:** 

O3—Co1—N1	92.51 (14)
O2^i^—Co1—O3	171.54 (13)
O3—Co1—O3^i^	78.42 (11)
O2^i^—Co1—N1	95.83 (14)
O3^i^—Co1—N1	170.41 (14)
O2^i^—Co1—O3^i^	93.33 (12)

Symmetry codes: (i) 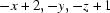; (ii) 
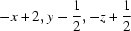
.

**Table 2:** Hydrogen-bond geometry (Å, °)

*D*—H⋯*A*	*D*—H	H⋯*A*	*D*⋯*A*	*D*—H⋯*A*
C8—H8⋯O5^iii^	0.93	2.55	3.295 (6)	138
C12—H12⋯O1^iv^	0.93	2.58	3.230 (7)	128

Symmetry codes: (iii) 
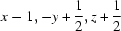
; (iv) 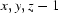.

**Fig. 1.**
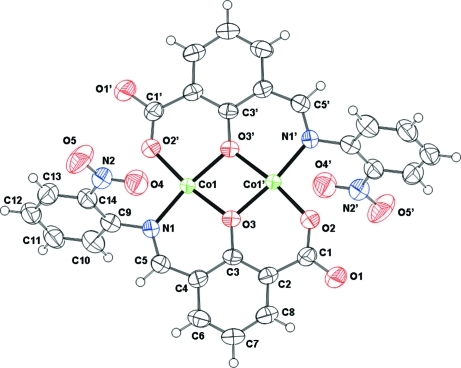
A view of the molecular structure of compound (I), showing the atom-labeling scheme. Atoms labeled (') are generated by the symmetry code -x + 2, -y, -z + 1.

**Fig. 2.**
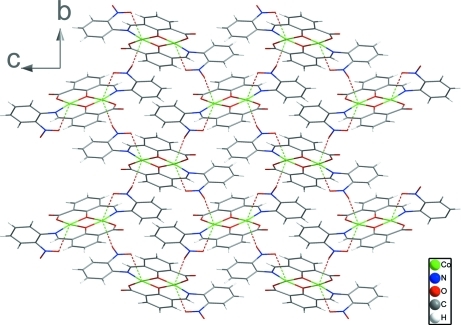
View along the a-axis of the two-dimensional network of complex (I).

**Fig. 3.**
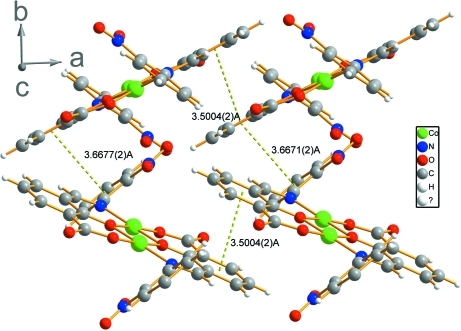
A view down the c axis of the π—π stacking interactions between the two-dimensional networks in compound (I).

**Fig. 4.**
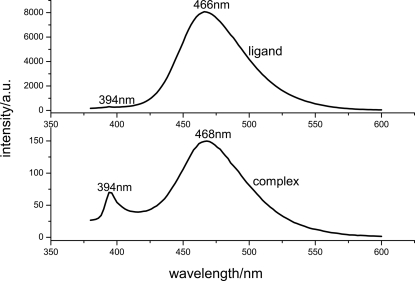
Emission spectra of the ligand and complex (I) in ethanol solution, λex = 351 nm.

## supplementary crystallographic information

### Comment

Weak intermolecular forces, such as hydrogen bonding, π—π stacking, dipole—dipole attractions, and van der Waals interactions, have been studied in depth and can be used in the design of molecular solids with specific supramolecular structures and functions (Zheng *et al.*, 2003). Numerous binuclear cobalt(II) complexes of Schiff bases have been studied (Tone *et al.*, 2007). To the best of our knowledge, 2-NA (2-nitroaniline) Schiff bases have not been reported up to now. As a result of our interest in the design of organic systems suitable for the assembly of supramolecular compounds, we have synthesized the Schiff base ligand H_2_CSNA, from the condensation reaction of 3-carboxylsalicylaldehyde with 2-nitroaniline, and its binuclear cobalt(II) complex, [Co_2_(CSNA)_2_], (I) [where CSNA^2-^ = 3-[(2-nitrophenyl)iminomethyl]-2-oxidobenzoate dianion].

Complex (I), a 2-NA Schiff base cobalt(II) complex, consists of centrosymmetric binuclear molecular units of [Co_2_(CSNA)_2_], as shown in Fig. 1. Selected bond distances and angles are given in Table 1. Each molecular unit contains two CSNA^2-^ anions and two Co^2+^ ions. The two CSNA^2-^ anions act as N1,O2,O3 tridentate ligands to chelate two Co^2+^ ions with the phenolate O-atoms (O3 and O3^i^: (i) = -*x* + 2, -*y*, -*z* + 1) as bridging atoms. The distance between the two metal centres is found to be 2.9950 (11) Å. These coordination bonds, forming a basal plane, are of normal strength with average bond length of Co—O 1.914 (3) Å, and Co—N 1.947 (4) Å.

The distances of the nitro group O-atoms O4 and O5^i^ [symmetry code: (i) -*x* + 2, -*y*, -*z* + 1] to atom Co1 are 2.8066 (44) Å and 2.8667 (44) Å, respectively. Calculated by the formula of s = exp[(r_o_-r)/B], (r = bond length, s = bond value) (Brown & Altermatt, 1985), the two bond values are 0.049 and 0.042. Hence, only weak interactions exist between atoms O4 and Co1, and Co1 and O5^i^ (Tabel 1). Thus every binuclear cobalt unit interacts with four adjacent ones through four nitro groups, forming a two-dimensional network (Fig. 2). The conformation of the ligand has changed obviously after coordination. It can be deduced that the free ligand might be a planar molecule because all the nonhydrogen atoms are in a conjugated system. In order to fulfil the intramolecular axial interaction of the nitro group, distortion has occurred between the two benzene rings of the ligand. In the binuclear molecular unit, the dihedral angle between the two benzene rings is 64.31 (13)°.

In the crystal structure there are C—H···O(nitro) interactions (Table 2), and relatively strong π···π stacking interactions [the distances between the centroids of the adjacent benzene rings are 3.5004 (2) Å, 3.6671 (2) Å and 3.6677 (2) Å] leading to the formation of a three-dimensional framework (Fig. 3).

The emission spectra of ethanol solutions of H_2_CSNA and complex (I) were measured at rt. As seen in Fig. 4, the results show that H_2_CSNA, exhibits two emissions at 394 nm and 466 nm (ν_ex_ = 351 nm). Measurement of the complex revealed two emissions at 394 nm and 468 nm (ν_ex_ = 351 nm). The similarity of the emissions bands of complex (I) and H_2_CSNA indicates that the luminescence emission of complex (I) may be assigned to the intraligand emission of the H_2_CSNA ligand (Li *et al.*, 2008).

In summary, an unprecedented 2-nitroaniline Schiff base complex, [Co_2_(CSNA)_2_], has been synthesized and structurally characterized. This successful synthesis provides a feasible and effective synthetic method for searching and exploring other novel 2-nitroaniline Schiff base complexes.

### Experimental

0.166 g (1.0 mmol) of 3-carboxylsalicylaldehyde and 0.138 g (1.0 mmol) of 2-nitroaniline were dissolved in 10 ml of methanol. The mixture was stirred at 60°C, and gradually a yellow precipitate (H_2_CSNA) was formed. 1 h later, 10 ml of a methanol solution of 0.08 g (2.0 mmol) NaOH, and 10 ml of a methanol solution of 0.285 g (1.2 mmol) CoCl_2_.6H_2_O were added to the H_2_CSNA solution, sequentially. After sirring for 1 h at 60°C the solution was allowed to cool to rt and then filtered. The filtrate was left to slowly evaporate at rt. After 10 d, blue crystals, suitable for X-ray structural analysis, were formed. FT/IR data for compound (I): 1634 cm^-1^(s, C=N), 1601 cm^-1 ^(s, benzene ring), 1579 cm^-1 ^(s, ν_as_(COO)), 1549 cm^-1^ (s, ν_as_(NO_2_)), 1360 cm^-1^ (s, ν_s_(COO)), 1299 cm^-1^ (s, ν_s_(NO_2_)).

### Refinement

All the H-atoms were included in calculated positions, with C—H distances constrained to 0.93 Å, and refined in the riding-model approximation, with *U*_iso_(H) = 1.2 *U*_eq_(parent C-atom).

### Crystal data

**Table:**

[Co_2_(C_14_H_8_N_2_O_5_)_2_]	*F*(000) = 692
*M**_r_* = 686.31	*D*_x_ = 1.877 Mg m^−^^3^
Monoclinic, *P*2_1_/*c*	Mo *K*α radiation, λ = 0.71073 Å
Hall symbol: -P 2ybc	Cell parameters from 1063 reflections
*a* = 8.3398 (6) Å	θ = 3.1–22.1°
*b* = 11.0454 (8) Å	µ = 1.44 mm^−^^1^
*c* = 13.3681 (9) Å	*T* = 296 K
β = 99.604 (1)°	Block, colourless
*V* = 1214.16 (15) Å^3^	0.21 × 0.16 × 0.11 mm
*Z* = 2	

### Data collection

**Table:**

Bruker SMART APEXII CCD area-detector diffractometer	2132 independent reflections
Radiation source: fine-focus sealed tube	1536 reflections with *I* > 2σ(*I*)
graphite	*R*_int_ = 0.045
φ and ω scans	θ_max_ = 25.0°, θ_min_ = 2.4°
Absorption correction: multi-scan (*SADABS*; Bruker, 2005)	*h* = −9→9
*T*_min_ = 0.752, *T*_max_ = 0.858	*k* = −11→13
6134 measured reflections	*l* = −15→14

### Refinement

**Table:**

Refinement on *F*^2^	Primary atom site location: structure-invariant direct methods
Least-squares matrix: full	Secondary atom site location: difference Fourier map
*R*[*F*^2^ > 2σ(*F*^2^)] = 0.045	Hydrogen site location: inferred from neighbouring sites
*wR*(*F*^2^) = 0.123	H-atom parameters constrained
*S* = 1.00	*w* = 1/[σ^2^(*F*_o_^2^) + (0.0704*P*)^2^] where *P* = (*F*_o_^2^ + 2*F*_c_^2^)/3
2132 reflections	(Δ/σ)_max_ < 0.001
199 parameters	Δρ_max_ = 0.63 e Å^−^^3^
0 restraints	Δρ_min_ = −0.34 e Å^−^^3^

### Special details

**Table:**

Geometry. Bond distances, angles etc. have been calculated using the rounded fractional coordinates. All su's are estimated from the variances of the (full) variance-covariance matrix. The cell esds are taken into account in the estimation of distances, angles and torsion angles
Refinement. Refinement of *F*^2^ against ALL reflections. The weighted *R*-factor *wR* and goodness of fit *S* are based on *F*^2^, conventional *R*-factors *R* are based on *F*, with *F* set to zero for negative *F*^2^. The threshold expression of *F*^2^ > σ(*F*^2^) is used only for calculating *R*-factors(gt) *etc*. and is not relevant to the choice of reflections for refinement. *R*-factors based on *F*^2^ are statistically about twice as large as those based on *F*, and *R*- factors based on ALL data will be even larger.

### Fractional atomic coordinates and isotropic or equivalent isotropic displacement parameters (Å^2^)

**Table:**

	*x*	*y*	*z*	*U*_iso_*/*U*_eq_	
Co1	0.98614 (6)	0.01580 (5)	0.38763 (4)	0.0353 (2)	
O1	0.6445 (5)	0.0607 (5)	0.7562 (3)	0.0940 (18)	
O2	0.8752 (4)	0.0241 (3)	0.7042 (2)	0.0581 (11)	
O3	0.8641 (3)	0.0427 (3)	0.4968 (2)	0.0452 (10)	
O4	1.0672 (5)	0.2394 (4)	0.3097 (3)	0.0765 (17)	
O5	1.1737 (5)	0.2946 (4)	0.1830 (4)	0.101 (2)	
N1	0.8176 (4)	0.0891 (3)	0.2876 (3)	0.0456 (12)	
N2	1.0763 (5)	0.2359 (4)	0.2183 (3)	0.0615 (17)	
C1	0.7269 (6)	0.0597 (5)	0.6879 (4)	0.0515 (17)	
C2	0.6501 (5)	0.1047 (4)	0.5856 (3)	0.0430 (16)	
C3	0.7180 (5)	0.0937 (3)	0.4962 (3)	0.0390 (14)	
C4	0.6304 (5)	0.1389 (4)	0.4043 (3)	0.0433 (16)	
C5	0.6823 (6)	0.1325 (4)	0.3083 (4)	0.0501 (17)	
C6	0.4790 (5)	0.1957 (4)	0.4036 (4)	0.0541 (19)	
C7	0.4134 (6)	0.2066 (4)	0.4893 (4)	0.0529 (19)	
C8	0.4995 (5)	0.1602 (4)	0.5784 (4)	0.0502 (17)	
C9	0.8350 (6)	0.0974 (4)	0.1838 (4)	0.0529 (17)	
C10	0.7265 (7)	0.0345 (5)	0.1114 (4)	0.0668 (19)	
C11	0.7394 (7)	0.0423 (5)	0.0094 (4)	0.069 (2)	
C12	0.8596 (7)	0.1094 (5)	−0.0233 (4)	0.065 (2)	
C13	0.9691 (7)	0.1698 (5)	0.0476 (4)	0.071 (2)	
C14	0.9579 (6)	0.1648 (4)	0.1501 (4)	0.0540 (17)	
H5	0.61030	0.16330	0.25350	0.0600*	
H6	0.42290	0.22650	0.34290	0.0650*	
H7	0.31340	0.24430	0.48820	0.0640*	
H8	0.45370	0.16660	0.63700	0.0600*	
H10	0.64530	−0.01270	0.13150	0.0800*	
H11	0.66480	0.00110	−0.03810	0.0830*	
H12	0.86670	0.11380	−0.09190	0.0780*	
H13	1.05180	0.21470	0.02670	0.0850*	

### Atomic displacement parameters (Å^2^)

**Table:**

	*U*^11^	*U*^22^	*U*^33^	*U*^12^	*U*^13^	*U*^23^
Co1	0.0287 (3)	0.0470 (4)	0.0303 (3)	0.0063 (3)	0.0052 (2)	0.0036 (3)
O1	0.062 (3)	0.174 (4)	0.049 (2)	0.029 (3)	0.018 (2)	−0.001 (3)
O2	0.048 (2)	0.083 (2)	0.0446 (19)	0.0159 (17)	0.0113 (15)	0.0061 (17)
O3	0.0358 (17)	0.0562 (18)	0.0441 (18)	0.0091 (14)	0.0080 (13)	0.0063 (15)
O4	0.072 (3)	0.091 (3)	0.065 (3)	−0.014 (2)	0.007 (2)	−0.004 (2)
O5	0.084 (3)	0.125 (4)	0.107 (4)	−0.035 (3)	0.052 (3)	0.003 (3)
N1	0.040 (2)	0.059 (2)	0.038 (2)	0.0039 (18)	0.0074 (16)	0.0094 (18)
N2	0.055 (3)	0.075 (3)	0.056 (3)	0.000 (2)	0.014 (2)	0.009 (2)
C1	0.046 (3)	0.067 (3)	0.043 (3)	0.005 (2)	0.012 (2)	−0.006 (2)
C2	0.035 (2)	0.047 (3)	0.048 (3)	−0.002 (2)	0.010 (2)	−0.005 (2)
C3	0.030 (2)	0.038 (2)	0.048 (3)	−0.0018 (18)	0.0033 (19)	−0.001 (2)
C4	0.036 (2)	0.046 (3)	0.049 (3)	−0.004 (2)	0.010 (2)	0.000 (2)
C5	0.040 (3)	0.060 (3)	0.049 (3)	0.003 (2)	0.004 (2)	0.010 (2)
C6	0.037 (3)	0.053 (3)	0.070 (4)	0.004 (2)	0.002 (2)	0.012 (3)
C7	0.033 (3)	0.059 (3)	0.067 (4)	0.006 (2)	0.009 (2)	−0.002 (3)
C8	0.037 (3)	0.057 (3)	0.058 (3)	−0.002 (2)	0.012 (2)	−0.012 (2)
C9	0.043 (3)	0.063 (3)	0.051 (3)	0.006 (2)	0.003 (2)	0.007 (2)
C10	0.060 (3)	0.076 (4)	0.060 (3)	0.001 (3)	−0.003 (3)	0.001 (3)
C11	0.067 (4)	0.084 (4)	0.050 (3)	0.014 (3)	−0.005 (3)	0.003 (3)
C12	0.066 (4)	0.083 (4)	0.045 (3)	0.022 (3)	0.004 (3)	−0.001 (3)
C13	0.071 (4)	0.083 (4)	0.064 (4)	0.023 (3)	0.027 (3)	0.016 (3)
C14	0.054 (3)	0.059 (3)	0.048 (3)	0.011 (2)	0.006 (2)	0.006 (2)

### Geometric parameters (Å, °)

**Table:**

Co1—O3	1.936 (3)	C4—C6	1.409 (6)
Co1—N1	1.947 (4)	C6—C7	1.355 (7)
Co1—O2^i^	1.874 (3)	C7—C8	1.383 (7)
Co1—O3^i^	1.929 (3)	C9—C10	1.395 (7)
O1—C1	1.231 (7)	C9—C14	1.401 (7)
O2—C1	1.281 (6)	C10—C11	1.388 (8)
O3—C3	1.341 (5)	C11—C12	1.375 (8)
O4—N2	1.237 (6)	C12—C13	1.374 (8)
O5—N2	1.196 (6)	C13—C14	1.390 (7)
N1—C5	1.298 (6)	C5—H5	0.9300
N1—C9	1.422 (7)	C6—H6	0.9300
N2—C14	1.457 (6)	C7—H7	0.9300
C1—C2	1.495 (7)	C8—H8	0.9300
C2—C3	1.411 (6)	C10—H10	0.9300
C2—C8	1.386 (6)	C11—H11	0.9300
C3—C4	1.412 (6)	C12—H12	0.9300
C4—C5	1.423 (7)	C13—H13	0.9300
			
Co1···O4	2.807 (4)	C3···C8^ix^	3.398 (6)
Co1···N2	3.488 (4)	C3···C13^iv^	3.347 (7)
Co1···O5^ii^	2.867 (5)	C4···C8^ix^	3.496 (6)
Co1···N2^ii^	3.404 (4)	C4···C2^ix^	3.583 (6)
Co1···C13^ii^	3.923 (6)	C4···C12^iv^	3.421 (7)
O1···C11^iii^	3.351 (7)	C5···O4	3.417 (7)
O1···C12^iii^	3.230 (7)	C6···C2^ix^	3.499 (6)
O2···N2^i^	3.057 (5)	C6···C1^ix^	3.421 (7)
O2···C9^i^	2.946 (6)	C7···C2^ix^	3.596 (6)
O2···C14^i^	3.030 (6)	C7···C3^ix^	3.509 (6)
O2···O3	2.766 (4)	C8···C3^ix^	3.398 (6)
O2···N2^iv^	3.126 (5)	C8···O5^x^	3.295 (6)
O2···O4^i^	2.961 (6)	C8···C4^ix^	3.496 (6)
O2···N1^i^	2.836 (5)	C11···C13^xi^	3.549 (8)
O3···O2	2.766 (4)	C11···O1^xii^	3.351 (7)
O3···O3^i^	2.444 (4)	C12···C4^viii^	3.421 (7)
O3···N1	2.806 (5)	C12···O1^xii^	3.230 (7)
O3···C13^iv^	3.334 (6)	C12···C3^viii^	3.510 (7)
O3···C5	2.891 (6)	C12···C13^xi^	3.437 (8)
O4···Co1	2.807 (4)	C12···C12^xi^	3.351 (8)
O4···N1	2.640 (5)	C13···C11^xi^	3.549 (8)
O4···C5	3.417 (7)	C13···Co1^vi^	3.923 (6)
O4···O2^i^	2.961 (6)	C13···C3^viii^	3.347 (7)
O5···C8^v^	3.295 (6)	C13···C12^xi^	3.437 (8)
O5···Co1^vi^	2.867 (5)	C13···O3^viii^	3.334 (6)
O1···H11^iii^	2.8100	C14···O2^i^	3.030 (6)
O1···H8	2.3700	C1···H12^iii^	3.0400
O1···H12^iii^	2.5800	C5···H10	2.8300
O2···H12^iii^	2.9100	C8···H5^iv^	3.0700
O4···H7^vii^	2.8800	C10···H5	2.6800
O4···H12^iv^	2.8100	H5···C10	2.6800
O5···H13	2.3400	H5···H6	2.2300
O5···H6^vii^	2.8200	H5···H10	2.5900
O5···H8^v^	2.5500	H5···C8^viii^	3.0700
N1···O3	2.806 (5)	H6···O5^xiii^	2.8200
N1···O4	2.640 (5)	H6···H5	2.2300
N1···N2	2.968 (5)	H7···O4^xiii^	2.8800
N1···C3	3.040 (6)	H7···H13^x^	2.3700
N1···O2^i^	2.836 (5)	H8···O1	2.3700
N2···Co1	3.488 (4)	H8···O5^x^	2.5500
N2···N1	2.968 (5)	H10···C5	2.8300
N2···Co1^vi^	3.404 (4)	H10···H5	2.5900
N2···O2^i^	3.057 (5)	H11···O1^xii^	2.8100
N2···O2^viii^	3.126 (5)	H12···O1^xii^	2.5800
C1···C6^ix^	3.421 (7)	H12···O2^xii^	2.9100
C2···C6^ix^	3.499 (6)	H12···C1^xii^	3.0400
C2···C4^ix^	3.583 (6)	H12···O4^viii^	2.8100
C2···C7^ix^	3.596 (6)	H13···O5	2.3400
C3···C12^iv^	3.510 (7)	H13···H7^v^	2.3700
C3···C7^ix^	3.509 (6)		
			
O3—Co1—N1	92.51 (14)	C4—C6—C7	121.5 (5)
O2^i^—Co1—O3	171.54 (13)	C6—C7—C8	118.1 (4)
O3—Co1—O3^i^	78.42 (11)	C2—C8—C7	123.9 (5)
O2^i^—Co1—N1	95.83 (14)	N1—C9—C10	119.0 (4)
O3^i^—Co1—N1	170.41 (14)	N1—C9—C14	123.2 (4)
O2^i^—Co1—O3^i^	93.33 (12)	C10—C9—C14	117.8 (5)
Co1^i^—O2—C1	130.1 (3)	C9—C10—C11	120.2 (5)
Co1—O3—C3	130.6 (2)	C10—C11—C12	121.7 (5)
Co1—O3—Co1^i^	101.58 (12)	C11—C12—C13	118.6 (5)
Co1^i^—O3—C3	127.7 (2)	C12—C13—C14	121.0 (5)
Co1—N1—C5	124.1 (3)	N2—C14—C9	122.8 (5)
Co1—N1—C9	121.2 (3)	N2—C14—C13	116.5 (4)
C5—N1—C9	114.7 (4)	C9—C14—C13	120.8 (5)
O4—N2—O5	122.0 (5)	N1—C5—H5	116.00
O4—N2—C14	119.2 (4)	C4—C5—H5	116.00
O5—N2—C14	118.6 (4)	C4—C6—H6	119.00
O1—C1—O2	121.3 (5)	C7—C6—H6	119.00
O1—C1—C2	117.9 (5)	C6—C7—H7	121.00
O2—C1—C2	120.8 (4)	C8—C7—H7	121.00
C1—C2—C3	125.1 (4)	C2—C8—H8	118.00
C1—C2—C8	117.1 (4)	C7—C8—H8	118.00
C3—C2—C8	117.8 (4)	C9—C10—H10	120.00
O3—C3—C2	121.6 (4)	C11—C10—H10	120.00
O3—C3—C4	119.4 (4)	C10—C11—H11	119.00
C2—C3—C4	119.0 (4)	C12—C11—H11	119.00
C3—C4—C5	125.2 (4)	C11—C12—H12	121.00
C3—C4—C6	119.8 (4)	C13—C12—H12	121.00
C5—C4—C6	115.1 (4)	C12—C13—H13	120.00
N1—C5—C4	128.1 (5)	C14—C13—H13	120.00
			
N1—Co1—O3—C3	0.1 (3)	O2—C1—C2—C3	11.4 (7)
N1—Co1—O3—Co1^i^	−176.86 (15)	O2—C1—C2—C8	−169.5 (4)
O3^i^—Co1—O3—C3	176.9 (4)	C1—C2—C3—O3	−1.7 (6)
O3^i^—Co1—O3—Co1^i^	−0.02 (14)	C1—C2—C3—C4	179.0 (4)
O3—Co1—N1—C5	−0.5 (4)	C8—C2—C3—O3	179.1 (4)
O3—Co1—N1—C9	−179.7 (3)	C8—C2—C3—C4	−0.2 (6)
O2^i^—Co1—N1—C5	178.1 (4)	C1—C2—C8—C7	179.9 (4)
O2^i^—Co1—N1—C9	−1.2 (3)	C3—C2—C8—C7	−0.9 (7)
N1—Co1—O2^i^—C1^i^	168.2 (4)	O3—C3—C4—C5	1.8 (6)
O3—Co1—O3^i^—Co1^i^	0.02 (14)	O3—C3—C4—C6	−178.2 (4)
O3—Co1—O3^i^—C3^i^	177.1 (3)	C2—C3—C4—C5	−179.0 (4)
Co1^i^—O2—C1—O1	166.4 (4)	C2—C3—C4—C6	1.1 (6)
Co1^i^—O2—C1—C2	−15.3 (7)	C3—C4—C5—N1	−2.4 (8)
Co1—O3—C3—C2	−179.9 (3)	C6—C4—C5—N1	177.6 (4)
Co1—O3—C3—C4	−0.7 (5)	C3—C4—C6—C7	−1.0 (7)
Co1^i^—O3—C3—C2	−3.7 (5)	C5—C4—C6—C7	179.0 (4)
Co1^i^—O3—C3—C4	175.5 (3)	C4—C6—C7—C8	0.0 (7)
Co1—N1—C5—C4	1.6 (7)	C6—C7—C8—C2	1.0 (7)
C9—N1—C5—C4	−179.1 (4)	N1—C9—C10—C11	179.2 (5)
Co1—N1—C9—C10	116.1 (4)	C14—C9—C10—C11	−1.7 (8)
Co1—N1—C9—C14	−63.1 (5)	N1—C9—C14—N2	−2.0 (7)
C5—N1—C9—C10	−63.3 (6)	N1—C9—C14—C13	−180.0 (4)
C5—N1—C9—C14	117.6 (5)	C10—C9—C14—N2	178.8 (5)
O4—N2—C14—C9	−1.4 (7)	C10—C9—C14—C13	0.9 (7)
O4—N2—C14—C13	176.6 (5)	C9—C10—C11—C12	1.2 (9)
O5—N2—C14—C9	−177.2 (5)	C10—C11—C12—C13	0.0 (9)
O5—N2—C14—C13	0.8 (7)	C11—C12—C13—C14	−0.8 (8)
O1—C1—C2—C3	−170.3 (5)	C12—C13—C14—N2	−177.7 (5)
O1—C1—C2—C8	8.9 (7)	C12—C13—C14—C9	0.4 (8)

Symmetry codes: (i) −*x*+2, −*y*, −*z*+1; (ii) −*x*+2, *y*−1/2, −*z*+1/2; (iii) *x*, *y*, *z*+1; (iv) *x*, −*y*+1/2, *z*+1/2; (v) *x*+1, −*y*+1/2, *z*−1/2; (vi) −*x*+2, *y*+1/2, −*z*+1/2; (vii) *x*+1, *y*, *z*; (viii) *x*, −*y*+1/2, *z*−1/2; (ix) −*x*+1, −*y*, −*z*+1; (x) *x*−1, −*y*+1/2, *z*+1/2; (xi) −*x*+2, −*y*, −*z*; (xii) *x*, *y*, *z*−1; (xiii) *x*−1, *y*, *z*.

### Hydrogen-bond geometry (Å, °)

**Table:**

*D*—H···*A*	*D*—H	H···*A*	*D*···*A*	*D*—H···*A*
C8—H8···O1	0.93	2.37	2.713 (7)	102
C8—H8···O5^x^	0.93	2.55	3.295 (6)	138
C12—H12···O1^xii^	0.93	2.58	3.230 (7)	128
C13—H13···O5	0.93	2.34	2.657 (7)	100

Symmetry codes: (x) *x*−1, −*y*+1/2, *z*+1/2; (xii) *x*, *y*, *z*−1.
